# Re: Quantitative assessment of bladder tissue properties using magnetic resonance fingerprinting: a pilot feasibility study in healthy volunteers

**DOI:** 10.1590/0100-3984.2025.0095

**Published:** 2025-10-21

**Authors:** Pradeep Tyagi

**Affiliations:** Research Professor, Department of Urology, University of Pittsburgh. E313 Montefiore Hospital, 3459 Fifth Ave, Pittsburgh, PA 15213. Email : tyagip@upmc.edu.

Dear Editor,

We read with interest the Correia et al. paper on magnetic resonance fingerprinting (MRF) of healthy human bladder wall by T_1_ and T_2_ relaxation times at 3-T scanner^(^1^)^. However, Table 1 values on bladder wall thickness ≤ 2 mm for three female subjects even when pre-void urine volume is 56 mL symbolizes errors in their technique, likely stemming from the use of T_2_ weighted scans for measuring bladder wall thickness^(^2^)^, contrary to the recommendations of Vesical Imaging Reporting and Data System (VI-RADS)^(^3^)^. The gender-neutral thickness of human bladder wall is authenticated to be ≥ 3 mm at the distension of ≥ 200–300 mL of urine or instilled agents^(^2^)^, which informs the recommended depth of 2 mm for intradetrusor injection of onabotulinumtoxinA. If the Table 1 values are correct, then intradetrusor injections would be causing bladder perforation in majority of patients. Authors could have avoided these missteps if they had chosen to read our extensive work on voxel-wise mapping of T_1_ relaxation times in rodent and human bladder^(^2^)^ which is freely available on the PubMed central. We achieved sub-millimeter resolution for variable flip angle T_1_ mapping by fast low angle shot in volume interpolated breath hold examination over a 17s breath hold at each flip angle. While authors speculated on the differences in T_1_ relaxation times of normal and diseased bladder wall, we^(^2^)^ were the first to measure T_1_ relaxation time in the bladder wall of interstitial cystitis/bladder pain syndrome at 3-T and others have reported higher T_1_ relaxation time in bladder wall of overactive bladder patients at 1.5-T scanner^(^4^)^. Despite numerous errors in reported values and lack of clarity on imaging sequence used for T_1_ mapping, authors certainly deserve applause for drawing attention to the potential use of MRF in urology.
